# Cardiac Biomarkers and Risk Scores in Relation with History of Atherosclerotic Cardiovascular Disease in Patients Admitted with COVID-19: The Experience of an Eastern European Center

**DOI:** 10.3390/jcm11195671

**Published:** 2022-09-26

**Authors:** Catalina Lionte, Victorita Sorodoc, Raluca Ecaterina Haliga, Cristina Bologa, Alexandr Ceasovschih, Oana Sirbu, Victoria Gorciac, Andrei-Costin Chelariu, Alexandra Stoica, Roxana Elena Tocila, Minerva Codruta Badescu, Irina-Iuliana Costache, Christiana Brigitte Sandu, Elisabeta Jaba, Laurentiu Sorodoc

**Affiliations:** 1 Internal Medicine Department, “Grigore T. Popa” University of Medicine and Pharmacy, 700115 Iasi, Romania; 2 Second Internal Medicine Clinic, “Sf. Spiridon” Emergency Clinical County Hospital, 700106 Iasi, Romania; 3 Rheumatology Department, Clinical Recovery Hospital, 700661 Iasi, Romania; 4 Hematology Department, Regional Institute of Oncology, 700483 Iasi, Romania; 5 Department of Cardiology, Cardiovascular Diseases Institute “Prof. Dr. George I.M. Georgescu”, 700503 Iasi, Romania; 6 III Internal Medicine Clinic, “St. Spiridon” County Emergency Clinical Hospital, 1 Independence Boulevard, 700111 Iasi, Romania; 7 Department of Internal Medicine (Cardiology), “Grigore T. Popa” University of Medicine and Pharmacy, 16 University Street, 700115 Iasi, Romania; 8 Statistics Department, Faculty of Economics and Business Administration, “Alexandru Ioan Cuza” University, 700506 Iasi, Romania

**Keywords:** N-terminal pro-B type natriuretic peptide, troponin I, Charlson comorbidity index, NEWS2 score, CoLACD score, atherosclerotic cardiovascular disease, COVID-19

## Abstract

Background: Data regarding the combined prognostic role of biomarkers and risk scores in relation with the history of atherosclerotic cardiovascular disease (ASCVD) in COVID-19 patients are lacking. Methods: The aim of this observational cohort study was to evaluate the combined prognostic value of N-terminal pro B-type natriuretic peptide (NT-pro BNP), troponin and risk scores in relation with ASCVD history in hospitalized COVID-19 patients. The primary composite endpoint was Intensive Care Unit (ICU) admission and death. Results: From April 2020 to June 2022, 1066 consecutive COVID-19 patients with available biomarkers upon admission were included. During a median follow-up period of 12 days, 176 patients (16.5%) died. Independent predictors of ICU admission and death in patients with ASCVD were NT-pro BNP (HR 2.63; 95% CI, 1.65–4.18) and troponin (HR 1.51; 95% CI, 1.13–2.03). In patients without ASCVD, only NT-pro BNP was predictive for the primary endpoint (HR 1.66; 95% CI, 1.10–2.53). This remained significant after adjustment for other relevant covariates (HR 3.54; 95% CI, 1.98–6.33) in patients with ASCVD and in patients without ASCVD (HR 1.82; 95% CI, 1.02–3.26). Conclusions: These data showed the combined prognostic accuracy of NT-pro BNP and troponin in relation with ASCVD history for ICU admission and death in COVID-19 patients.

## 1. Introduction

Since the beginning of the novel coronavirus disease 2019 (COVID-19) pandemic in March 2020, multiple studies trying to understand the mechanism and consequences of this viral disease have been published. These were primarily focused on patients admitted to the hospital with critical forms of COVID-19 [1,2,3,4]. COVID-19 cases are escalating morbidity in patients with cardiovascular diseases [5]. One of the main consequences of COVID-19 disease is cardiovascular damage (i.e., myocarditis, arrhythmias, acute coronary syndrome, and pulmonary embolism) [6,7]. The infection affects cardiac muscle integrity, fibrinogen pathways, redox homeostasis and induces a break in atheromatous plaques, which aggravates myocardial injury and dysfunction [8]. Myocardial injury is associated with a higher risk for adverse events in COVID-19 patients, and was described in over 50% of intensive care unit (ICU) patients and between 10% and 45% in hospitalized non-ICU patients [9].

Studies have proven the link between cardiac biomarkers in critically ill COVID-19 patients and in-hospital mortality [1,2,3]. Myocardial injury is a common complication among hospitalized COVID-19 patients [2,4], and accordingly, cardiac biomarkers in these patients are changing and were shown to predict a poor outcome [10,11,12]. However, cardiac biomarkers fail to differentiate between myocarditis secondary to infection of cardiac tissue and myocardial injury related to ischemic heart disease [7]. Still, information regarding risk scores, biomarkers and outcome prediction for patients with mild and moderate COVID-19 disease in relation with a history of atherosclerotic cardiovascular disease (ASCVD) is scarce. 

Cardiac biomarkers proved to be useful for risk stratification and prognosis in the case of heart failure (HF) [13], coronary artery disease (CAD) or acute coronary syndrome (ACS) [14,15], pulmonary embolism [16], and community-acquired pneumonia [17]. Some risk scores, such as the Charlson comorbidity index and CoLACD (COVID-19, Lymphocyte ratio, Age, CCI score, Dyspnea), a novel COVID-19 mortality index [18] or the National Early Warning Score 2 (NEWS2) [19] were developed to identify inpatient deterioration or mortality from the SARS-CoV2 infection. To our knowledge, studies involving the role of cardiac biomarkers and risk scores in relation with history of ASCVD in COVID-19 patients are lacking. In our study, we focused on patients with medical emergencies and associated mild and moderate forms of COVID-19 admitted in a tertiary Eastern European emergency hospital.

Thus, the main objective of our study was to observe and confirm the relationship between cardiac biomarkers and risk scores with a composite outcome consisting of ICU admission and in-hospital mortality of mild to moderate COVID-19 patients, in relation with the history of ASCVD. Atherosclerotic cardiovascular disease was defined by the history of one or more of the following: CAD (i.e., chronic coronary syndrome), cerebrovascular atherosclerosis, aortic atherosclerosis, renal artery atherosclerosis and peripheral arterial diseases [20]. As a secondary objective, we assessed the predictive role of biomarkers on the occurrence of major adverse cardiovascular events (MACEs), the need for mechanical ventilation (MV), and other complications during hospital admission in patients with mild or moderate forms of COVID-19, defined according to the guidelines [21].

## 2. Materials and Methods

### 2.1. Study Design

This study is reported in line with the Strengthening the Reporting of Observational Studies in Epidemiology (STROBE) guidelines for observational studies.

This was a prospective cohort study aiming to assess the prognostic role of cardiac biomarkers and risk scores in patients hospitalized for a medical emergency with associated mild or moderate COVID-19, in relation with the presence or absence of the history of ASCVD, for the need for ICU admission and in-hospital mortality, MACEs, MV and other in-hospital complications. We obtained data on the entire cohort, and we aimed to confirm that the history of ASCVD is a risk factor for higher mortality and increased ICU admission rates. We also tried to define the risk factors that can worsen COVID-19 prognosis in a group of patients that has an embedded higher risk because of their existing ASCVD. Mild COVID-19 disease was defined as signs and symptoms of COVID-19 (fever, coughing, loss of smell, loss of taste) but absence of shortness of breath, dyspnea or abnormal chest imaging, and moderate COVID-19 was defined as evidence of lower respiratory disease during clinical assessment or imaging and an oxygen saturation ≥94% on room air [21].

The primary outcome was a composite endpoint, defined as ICU admission and in-hospital mortality in the subgroups of patients with or without a history of ASCVD. The secondary outcome was the occurrence of MACEs, the need for MV, and other in-hospital complications in patients with documented COVID-19, in relation with the history of ASCVD.

### 2.2. Population and Setting

Twelve hundred nineteen patients aged over 18 years, who were admitted for a medical emergency between 1 April 2020 and 30 June 2022, and had confirmation of SARS-CoV-2 infection by RNA reverse-transcriptase polymerase chain reaction (RT-PCR) assay were included in this study. Patients with multiple readmissions during the study period were evaluated as a single presentation. Patients with definite clinical outcomes (i.e., discharged or deceased) were followed up until 15 July 2022, and 20 patients who requested discharge against medical advice, and for whom we had no information about clinical endpoints were excluded. We also excluded 30 patients with COVID-19 who were transferred to other hospitals for specific procedures, and 103 patients with missing information on cardiac biomarkers or RT-PCR assay. Finally, 1066 patients were included in the analyses. The patients were followed up only during hospitalization. A detailed flow diagram of our sample is presented in Supplementary Figure S1.

ASCVD was recorded as follows: CAD in 212 patients (19.9%), cerebrovascular atherosclerosis in 91 patients (8.5%), aortic aneurisms in 19 patients (1.8%) and peripheral artery disease in 105 patients (9.8%). There were 66 patients with atherosclerotic lesions localized in different vascular territories, but they were counted once, based on the main site of the disease. This study was approved by our Institutional Review Board, and an individual written informed consent was obtained from the patient or next of kin, in the case of patients with an altered mental status.

### 2.3. Variables and Data Collection

For data extraction, we recorded demographics, vaccination status, vital signs, body mass index (BMI) and comorbidities. Comorbidities were defined by their recording in the medical chart and consisted of diabetes mellitus (DM), hypertension (HT), chronic heart failure (HF), defined as history of previous congestive decompensation or diagnosis of left ventricular systolic dysfunction (LVEF < 40%), chronic coronary syndrome (CCS), acute coronary syndrome (ACS), atherothrombotic or cardioembolic stroke, vascular disease, chronic obstructive pulmonary disease (COPD), bronchial asthma, hepatic disease, hematologic and oncologic disease, or an estimated glomerular filtration rate of <50 mL/min for chronic kidney disease (CKD). The Charlson comorbidity index (CCI) assessed according to the scoring system established by Charlson et al. [22] was calculated for each patient. A hypertensive (HT) emergency was defined as grade 3 hypertension associated with encephalopathy, acute heart failure, acute myocardial ischemia or acute deterioration in kidney function which required immediate intervention with intravenous therapy, as recommended by the guidelines [23]. A metabolic emergency was defined either as an acute metabolic emergency in diabetes mellitus [24] or as acid-base disorders and dyselectrolytemia requiring parenteral therapy [25]. Another cardiovascular emergency was defined as a decompensation of a chronic heart condition, requiring medical intervention (i.e., tachy or bradyarrhythmia, valve disease, cardiomyopathies, myocarditis, pericarditis, chronic HF, etc.). Additionally, the NEWS2 score [26], the CCI [22] and the CoLACD [18] scores were calculated from the clinical records. Biochemistry and hematology results upon admission, first electrocardiogram (ECG) recorded, in-hospital clinical course and complications, treatment and outcomes were extracted from the index hospital admission using a standardized electronic data form. These investigations were repeated during hospital stay at the indication of the attending physician, in relation with the patients’ evolution. The results were obtained using PATHFAST Cardiac Biomarker Analyzer (LSI Medience Corporation, Tokyo, Japan), Sysmex XT-4000i—Automated Hematology Analyzer (Sysmex Corporation, Tokyo, Japan), and ARCHITECT c16000 Clinical Chemistry Analyzer (Abbott Laboratories, Abbot Park, IL, USA). The PATHFAST NTproBNP and hs-cTnI assay principle is based on chemiluminiscence enzyme immune assay and *MAGTRATION**^®^** methodology, and the manufacturer reference interval is <15–128 pg·mL for the NTproBNP, and 0–29 ng/L for hs-cTnI, with the 99th percentile of URL of 29.7 ng/L for males and 20.3 ng/L for females.

### 2.4. Statistical Methods

Categorical variables are summarized as percentages and continuous variables are defined as the number of non-missing observations, the mean and standard deviation (SD), or the median and interquartile range [IQR]. Variables were compared between outcome groups (ICU admission and in-hospital mortality, MACEs, MV, and other in-hospital complications) using Mann–Whitney or Chi-square tests, as appropriate. For the analysis as a continuous variable, the concentrations of D-dimer, NT-pro BNP and hs-TnI assessed during hospitalization were log transformed. Missing data were excluded pairwise where applicable. We performed Cox proportional hazards regression to estimate the association between cardiac biomarkers and all-cause mortality adjusting for age, sex, CCI, NEWS2 score ≥5, CoLACD score, and comorbidities (including HF, CKD, DM) in the subgroups of patients with and without a history of ASCVD. To tightly control the confounding factors from the baseline disease severity, we further adjusted for virus strain, baseline oxygen saturation >90%, BMI, creatinine, and high-sensitivity C-reactive protein (all continuous). Subsequent adjustment with hs-TnI and D-dimers was performed in patients with and without history of ASCVD. The time to events was denoted as the days from the moment of disease onset to death or hospital discharge. Kaplan–Meier survival curves for the composite endpoint were plotted, and the log-rank test was computed to assess differences between groups of patients based on the history of ASCVD, quartiles of NT-pro BNP and abnormal hs-TnI. Receiver-operating curves (ROC) were built to analyze the diagnostic performance of the multivariate models for prediction of ICU admission and in-hospital mortality. A statistical test was significant when *p* value was <0.05. All *p* values are the results of 2-tailed tests. Statistical analyses were performed using SPSS software version 22 (IBM Corp, Chicago, IL, USA).

## 3. Results

We included 1066 patients with a median age of 70 years, 542 males (51.3%) with confirmed mild or moderate COVID-19. The majority of patients were infected with the Alpha and Delta variants (47.7% and 32.7%), while the Beta and Omicron viral strains were involved in 11.3% and in 8.3%, respectively, of all patients. Only 72 patients (6.8%) included in our cohort had been vaccinated prior to hospital admission (13.6% of the subgroup without history of ASCVD and 21.2% from the subgroup with ASCVD, *p* = 0.046). Three hundred and twenty-four patients of the entire cohort (74.1%) had a NEWS2 score >5. A significantly lower number of vaccinated patients had a NEWS2 score >5: 45 patients (62.5%) versus 279 non-vaccinated patients (76.4%, *p* 0.018). Baseline characteristics of the cohort are presented in Table 1. We recorded 361 patients with history of ASCVD (33.9%) and 705 patients without ASCVD (66.1%), Table 1. We recorded 77 patients (21.3%) transferred to ICU who did not survive, among patients with a history of ASCVD, and 99 patients (14.0%) with need of ICU therapy and in-hospital death among patients without ASCVD (Supplementary Figure S1).

Based on the SARS-CoV2 strain involved, only the Delta variant was significantly correlated with mortality in patients with history of ASCVD (21.0%, vs. 33.3%, *p* =0.017). The presence of HF was significantly associated with the main composite outcome in patients with ASCVD (Table 1). Moreover, the history of CKD significantly influenced the composite main outcome, both in patients without ASCVD and in patients with ASCVD (Table 1).

The hospital admission for a HT emergency, according to the European Society of Cardiology guidelines recommendation [27], was significantly correlated with a poor outcome in patients with ASCVD (Table 2). Among the 572 patients admitted with a HT emergency, we recorded 32.5% patients with associated acute deterioration in renal function, 26.9% patients with acute heart failure, 17.1% patients with associated encephalopathy, 17% patients with malignant hypertension and funduscopic changes, 5.6% patients with associated acute myocardial injury and 0.9% patients with secondary hypertension. A total of 439 patients (41.2%) who developed severe or critical COVID-19 required MV for a median period of 2 days (range 1–46 days), 869 patients (82.3%) developed in-hospital complications, and MACEs were recorded in 73 patients (6.8%); 176 patients (16.5%) died during hospitalization. The main complications recorded were: acute respiratory failure (388 cases, 36.4%), secondary infections (196 patients, 18.4%), multiple organ dysfunction (51 cases, 4.8%), venous thromboembolism (48 patients, 4.5%), acute liver and kidney injury (40 cases, 3.8%; 38 patients, 3.6%) and shock (39 patients, 3.7%). Twenty-five patients (2.3%) developed an acute coronary syndrome (ACS) during hospitalization. Of note, in the entire cohort, 25 patients (2.3%) developed acute pulmonary embolism (PE). PE was the main cause of death in 3 patients (0.5%) without ASCVD, and in 2 patients (0.5%) among those with ASCVD.

The median number of days spent in hospital was 12 (range 1–59 days). 698 patients (65.5%) received symptomatic treatment. Anticoagulation therapy administered either in prophylactic or therapeutic dose (Table 2) significantly influenced survival in both subgroups of patients. Antiviral therapy did not show a significant effect on the main composite outcomes in our cohort, while immunomodulator therapy significantly influenced the main composite outcome only in patients with a history of ASCVD (Table 2). Although a low number of patients in our cohort had been vaccinated, we observed that vaccination protected against ICU admission and/or death, since it was recorded in 34 vaccinated patients (12.9%) versus 38 non-vaccinated patients (22.0%, *p* = 0.017).

### 3.1. NT-proBNP and Risk Scores in Relation with the Outcomes

**Figure 1:**
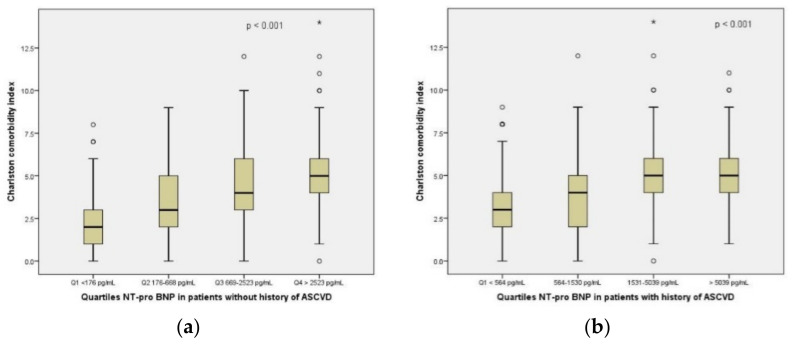
CCI is correlated with NT-pro BNP quartiles, based on the history of ASCVD: (**a**) CCI score and NT-pro BNP quartiles in patients without history of ASCVD; (**b**) CCI score and NT-pro BNP quartiles in patients with history of ASCVD. °, represent outliers; *, represent extreme values.

NT-pro BNP was significantly increased in patients with ASCVD as compared to patients without ASCVD (a median of 1530 pg/mL vs. 668 pg/mL, *p* < 0.001). There were also significantly higher values of NT-pro BNP recorded in patients with ASCVD exposed to Alpha, Beta and Delta strains (Supplementary Table S1). NT-pro BNP quartiles were significantly correlated with the CCI score in both groups of patients, with and without ASCVD (Figure 1a,b). The CoLACD and NEWS2 risk scores were not significantly correlated with the quartiles of NT-pro BNP, irrespective of the history of ASCVD.

Additionally, quartiles of NT-pro BNP were significantly correlated with hs-TnI in patients with and without a history of ASCVD (Supplementary Figure S2a,b). We analyzed the correlation between NT-pro BNP quartiles with D-dimer levels based on the history of ASCVD. We observed a significant correlation between D-dimer levels and quartiles of NT-pro BNP in patients without ASCVD (Supplementary Figure S3a), but this was not confirmed for the patients with ASCVD (Supplementary Figure S3b).

None of the risk scores analyzed showed a significant correlation with the main outcome in multivariate analysis. Hypertensive emergency as the reason of admission and the viral strain Delta were significantly correlated with the main composite outcome in the multivariate model (Table 3).

**Figure 2:**
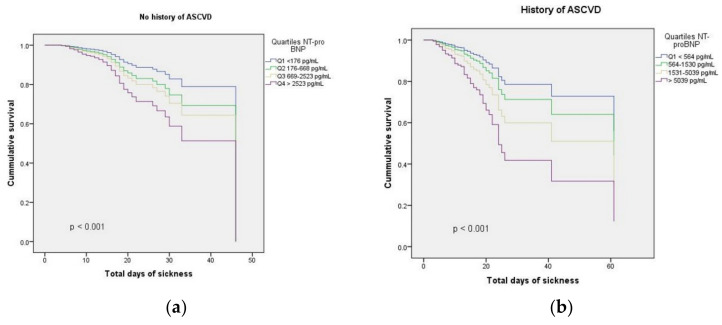
Kaplan–Meier survival curves regarding all-cause mortality according to the admission NT-pro BNP quartiles: (**a**) NT-pro BNP quartiles in patients without history of ASCVD; (**b**) NT-pro BNP quartiles in patients with history of ASCVD.

A multivariable Cox model confirmed that NT-pro BNP was independently associated with in-hospital death, after adjustment for all relevant confounders both in patients with a history of ASCVD: hazard ratio (HR) 2.63, 95% confidence interval (CI) 1.65–4.18, per logarithmic unit, and in patients without a history of ASCVD (HR 1.66, 95% CI 1.10–2.53, per logarithmic unit), Table 3, Figure 2.

We performed complementary analyses in order to consider further adjustment for other biomarkers available. Even after adjustment for hs-TnI (Supplementary Table S2) and D-dimer (Supplementary Table S3), NT-pro BNP remained independently associated with the main outcome: HR 1.62 (1.08–2.43) in patients without ASCVD, and HR 2.69 (1.68–4.29) in patients with a history of ASCVD; HR 1.69 (1.09–2.63) in patients without ASCVD, and HR 3.34 (1.89–5.88) in patients with a history of ASCVD.

We tested NT-pro BNP in relation with the secondary outcomes, and it showed a strong correlation with MACEs in both groups of patients, but had no significant influence on the other secondary outcomes (Supplementary Table S4).

### 3.2. hs-TnI and Risk Scores in Relation with the Outcomes

We observed a significant correlation between hs-TnI upon admission and the composite outcome (Supplementary Figure S4a) both in patients with no history of ASCVD, and in patients with ASCVD (Supplementary Figure S4b). However, hs-TnI was significantly higher only in patients with ASCVD infected with Alpha strain, compared to patients without a history of ASCVD (Supplementary Table S1). In patients with ASCVD, the median hs-TnI upon admission was 9 ng/L in survivors and 23.1 ng/L in patients who were admitted to ICU and died (*p* =0.003). In patients without ASCVD, the median hs-TnI upon admission, although significantly higher in patients with a poor outcome versus survivors (15.9 vs. 5.9 ng/L, *p* =0.001), was lower than the values recorded in patients with ASCVD. Although these initial median values of hs-TnI are below the 99th percentile of the upper reference limit (>29 ng/L), as recommended by the manufacturer, we documented myocardial injury, defined as patients with hs-TnI levels above the 99th percentile of URL and new ECG changes of the ST segment or T wave [28] in both groups of patients: 32 survivors (7.8%) vs. 56 non-survivors (18.9%) in the group without a history of ASCVD, and 29 survivors (14.4%) vs. 40 non-survivors (24.1%) in the group with a history of ASCVD. The presence of myocardial injury significantly correlated with the ICU admission and death in both groups with no history of ASCVD (*p* < 0.001), and with ASCVD (*p* =0.007). However, new ST/T changes were significantly correlated with the main composite outcome only in the subgroup of patients without ASCVD (*p* =0.042). Moreover, in the entire cohort, we did not find any correlation between ECG changes upon admission, in terms of QRS complex amplitude and the outcomes. There was no correlation between the risk scores and abnormal levels of hs-TnI, irrespective of the history of ASCVD.

**Figure 3:**
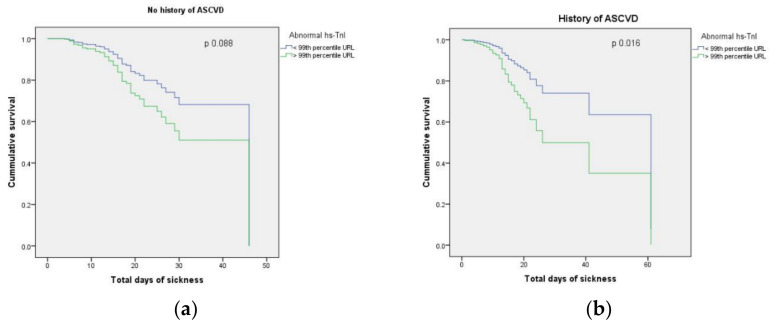
Kaplan–Meier survival curves regarding all-cause mortality according to the admission abnormal high-sensitivity troponin I (hs-TnI): (**a**) In patients without history of Atherosclerotic Cardiovascular Disease (ASCVD); (**b**) In patients with history of ASCVD; URL, upper reference limit.

High-sensitivity TnI levels were significantly associated with evolution towards critical COVID-19 and death, but only in patients with a history of ASCVD, as shown in Table 4 and Kaplan–Meier survival curves in Figure 3.

Even after adjustment for D-dimer, hs-TnI remained independently associated with the main composite outcome [HR 1.63 (1.14–2.33), *p* =0.008] in patients with a history of ASCVD.

Significant correlations with all the secondary outcomes were recorded for hs-TnI in patients with a history of ASCVD. However, hs-TnI was significantly correlated only with MACEs in patients without a history of ASCVD (Supplementary Table S5).

### 3.3. Predictive Capacity of Biomarkers in Relation with the Main Outcome

To observe the increment in discrimination among the models including different biomarkers, we assessed the predictive role for the main composite outcome in a model including classical cardiovascular risk factors (model 1), to which we added NT-pro BNP in patients with COVID-19 (model 2) and hs-TnI (model 3). Then, we added D-dimers to model 2 (model 4), and finally hs-TnI to model 4 (model 5, Supplementary Table S6). The model which proved to have the best discriminative power for ICU admission and mortality in COVID-19 patients with ASCVD was the one which included NT-pro BNP, hs-TnI and D-dimers (AUC 0.751; 95% CI: 0.69–0.82), Supplementary Figure S5. NT-pro BNP outperformed hs-TnI since it was strongly and independently correlated with the main outcome both for patients without a history of ASCVD, and in patients with ASCVD, while troponin showed a predictive capacity for this outcome only in patients with a history of ASCVD (Supplementary Table S6).

## 4. Discussion

This study provides novel data regarding the relationship between cardiac biomarkers, risk scores and mortality in patients with mild and moderate forms of COVID-19, in relation with the history of ASCVD. First, this is the largest study to include patients with mild and moderate COVID-19 tested for cardiac biomarkers and risk scores to analyze the impact of the previous history of ASCVD on the severity of the disease and mortality. We decided to focus on the population with a history of ASCVD because although COVID-19 is primarily a respiratory disease, it also affects the cardiovascular system, especially through endothelial dysfunction. The virus enters the host cells (macrophages, type 2 pneumocytes, pericytes, cardiac myocytes and endothelial cells) via transmembrane ACE2, causing inflammation and affecting multiple organs. After infecting the endothelial cells and pericytes, SARS-CoV2 could cause macrovascular and microvascular dysfunction. Moreover, the cytokine storm caused by immune over-reactivity can potentially destabilize atherosclerotic plaques, leading to ACS [6]. In our cohort, a low proportion of patients developed an ACS during hospitalization. However, cardiac biomarkers were detected at high levels especially in non-survivors, both in the group of patients without a history of ASCVD and in patients with ASCVD. We can ascribe endothelial dysfunction for this development.

Studies proved that we can use cardiac biomarkers (i.e., NT-pro BNP, hs-TnI) to predict mortality in critically ill COVID-19 patients [14,29,30]. Moreover, there is a significant association between COVID-19 disease severity and levels of cardiac troponin, although mild elevations in cardiac hs-Tn rather reflect preexisting cardiovascular disease, or acute injury related with COVID-19 [31]. Our analysis supports these findings, proving that they also apply to the population with mild and moderate forms of COVID-19, with a history of ASCVD. Thus, these two cardiac biomarkers can be used to stratify mortality risk in this population and the potential evolution to a severe form of disease, with the need for ICU therapy. It was already proved that in COVID-19 patients with ACS cardiac biomarkers are useful as predictors of progression to a severe COVID-19 form [4]. However, the low percentage of patients with ACS in our cohort could not have influenced our results in patients with mild or moderate COVID-19.

Secondly, we also observed a good predictive value of NT-pro BNP for the evolution towards a critical disease and death in patients without a history of ASCVD. Patients without ASCVD and higher NT-pro BNP levels were older, had more cardiac and non-cardiac comorbidities, presented a low oxygen saturation upon presentation, and the severity of the disease assessed using NEWS2 and CoLACD scoring systems was notable. Our findings are in line with the results of several investigations which demonstrated that the clinical outcomes in patients with SARS-CoV-2 infection are closely related to the burden of associated comorbidities [32,33,34].

Thirdly, the present study results support the hypothesis that cardiac biomarkers, especially NT-pro BNP and hs-TnI, are highly associated with ICU admission and mortality in hospitalized patients with ASCVD and non-severe COVID-19. We confirmed a strong and independent association of elevated NT-pro BNP and troponin levels with the ICU admission and mortality in patients with mild or moderate COVID-19 and a history of ASCVD. However, in our multivariate model for patients without a history of ASCVD, troponin could not predict a composite outcome of admission to ICU and hospital mortality. These results are in line with other reported studies which included patients with COVID-19 with and an Acute Physiology and Chronic Health Evaluation II (APACHE II) score over 13 admitted to ED [35], and consecutive unselected hospitalized COVID-19 patients, where hs-TnT, in a multivariate model adjusted for clinical variables and NEWS score, failed to predict a similar composite outcome [36].

Moreover, NT-pro BNP appeared to be a valuable biomarker in predicting ICU admission and death also for patients without ASCVD. The circulating levels of NT-pro BNP and hs-TnI can be differently affected by the mechanisms involved in cardiac dysfunction and/or injury, and the increase of both biomarkers can be a result of powerful stressor mechanisms which cause relevant alterations of cardiac function (resulting in increased natriuretic peptides), significant damage of cardiomyocytes and myocarditis, or perhaps direct effects of SARS-CoV2 on microvasculature (resulting in increased troponins) [7,37,38]. The incidence of myocardial injury increases with greater disease severity and ARDS occurrence [38]. However, for a significant number of COVID-19 patients, myocardial injury is chronic, and not directly related with COVID-19 [9].

Another possible explanation of NT-pro BNP elevated levels is the unmasking of subclinical HF in patients with ASCVD, or exacerbation of a pre-existing HF, as a result of increased metabolic demands of COVID-19 [6]. Higher circulating levels of troponin in patients with and without ASCVD might be a consequence of direct SARS-CoV-2 effects on cardiomyocytes [39] or through up-regulation of ACE2 in the heart and coronary vessels, hypoxia and immune mechanisms of myocardial inflammation [6,40]. Cardiac biomarkers are increased according to the severity of COVID-19 [10]. Furthermore, we observed that hs-TnI and D-dimer improved the prognostic accuracy of NT-pro BNP for the outcomes analyzed, which proved to be a strong predictor for ICU admission and death, even in patients without a history of ASCVD. NT-pro BNP reflects hemodynamic deterioration, myocardial wall stress, myocardial ischemia, alterations in volume loading, and renal function. Thus, NT-pro BNP elevation reflects more than an extensive cardiovascular injury in COVID-19 patients [10]. Moreover, inflammation, impairment of cardiac function in the case of acute heart failure, interactions with ACE2 might be responsible for higher circulating levels of natriuretic peptides in COVID-19 cases [31]. However, future research on the pathophysiological mechanisms linking increase in cardiovascular biomarkers and COVID-19 is needed.

Fourthly, we show for the first time that adding the D-dimer and hs-troponin I improves the prognostic accuracy of NT-pro BNP for the outcomes analyzed, as opposed to a model which included traditional cardiovascular risk factors. Indeed, it was proved that standard cardiovascular risk factors are poor predictors of developing myopericarditis with COVID-19 [41]. However, NT-pro BNP is also important for diagnosis of myopericarditis associated with COVID-19, as the patients do not always have typical symptoms [42]. The correlation observed between quartiles of NT-pro BNP and D-dimer levels was noticed in other studies, which included COVID-19 patients with history of HF [29]. However, we showed for the first time a correlation between these two biomarkers in patients without ASCVD, which can be explained by the fact that both are biomarkers of comorbidity and inflammation in COVID-19, and both proved their role for prognostic stratification in pulmonary embolism, pneumonia, heart failure and COVID-19 patients [16,17,43,44,45]. Moreover, due to the role of endotheliitis and VTE in COVID-19, elevated D-dimers, which quantify activated coagulation, represent a prominent feature in COVID-19 patients [6]. It is unsurprising that a severe inflammatory disease can trigger cardiac complications in low risk COVID-19 patients without a history of ASCVD, but also in patients with pre-existing cardiovascular risk factors [42].

Zwaenepoel et al. concludes that hs-TnI and NT-proBNP outperform other routinely used biomarkers (C-reactive protein, D-dimer and ferritin), as well as clinical indices of disease severity in ICU, such as total SOFA, respiratory SOFA and P/F ratio in critically ill COVID-19 patients [46]. Our data suggest that these cardiac biomarkers have similar utility in the case of mild and moderate forms of COVID-19. Consistent with previously published data, patients with elevated levels of hs-TnI and higher NT-proBNP developed MACEs more often during hospitalization in our cohort. The presence of cardiac injury, depicted by early measurement of hs-TnI and NT-proBNP, proved to be a predictor of severe complications in COVID-19 infection and should prompt advanced treatments, as needed [10].

All the clinical scores we used for prognosis stratification in our cohort (CCI, NEWS2 and CoLACD scores) proved to correlate with ICU admission and in-hospital mortality in patients without ASCVD, while only NEWS2 was correlated with ICU admission and death in patients with a history of ASCVD. Our results are in line with other studies, which reported that an age adjusted CCI is predictive for mortality and invasive mechanical ventilation [47], and NEWS2 score can identify inpatients deterioration [19]. The CoLACD score proved to be useful as a mortality predictor in COVID-19 patients [18]. Our study finds an association of CoLACD score with ICU admission and death only for patients without ASCVD. Moreover, we showed a significant correlation between increasing levels of NT-pro BNP, with CCI.

Lampert et al. suggested that low amplitude of QRS complex is an independent predictor of mortality in hospitalized patients with COVID-19 [48]. Our analysis did not find a significant correlation between the amplitude of QRS complex, or other ECG changes described by these authors, (such as QRS morphology due to changes in rhythm origin or new bundle branch block) and in-hospital mortality. Our results can be explained by the fact that our cohort included patients with mild or moderate COVID-19, whereas the original study included critically ill patients.

In contrast with the results of Mountantonakis et al. showing that new-onset AF was an independent predictor of in-hospital mortality in COVID-19 patients [49], our analysis did not record a correlation between new-onset AF and ICU admission and in-hospital mortality neither in patients with COVID-19 and ASCVD, nor in patients without history of ASCVD. A possible explanation could be the low number of patients with this diagnosis in our cohort (1.4%), as opposed to the above-mentioned study.

We noticed that in patients with ASCVD, a hypertensive emergency upon admission and a history of HF were significantly associated with ICU admission and death. Our data are in line with the observations of Lippi et al., who pointed out that hypertension might be linked an up to 2.5-fold greater significant risk of lethal COVID-19, particularly with older individuals [50]. However, in the final Cox regression model, a hypertensive emergency remained predictive for ICU admission and death only in patients with a history of ASCVD and mild-to-moderate COVID-19. Nevertheless, after adjusting for other relevant covariates, the history of HF did not reach statistical significance to be identified as an independent predictor for ICU admission and mortality for this population. This observation is supported by the results of Rey et al., who could not identify HF as an independent predictor of mortality in COVID-19 patients, even though HF patients can develop an acute decompensation after COVID-19 diagnosis [51].

Regarding the treatment regimen, our analysis showed that antiviral therapy did not correlate with in-hospital mortality, irrespective of the history of ASCVD, thus supporting the existent data [52,53,54]. Studies have shown that dexamethasone reduced mortality in hospitalized COVID-19 patients receiving oxygen, especially in those with critical forms receiving mechanical ventilation [55]. In our cohort which included only non-critically ill COVID-19 patients, we did not observe a significant increase of survival, irrespective of the history of ASCVD, after corticotherapy administration. Alternatively, significant inverse correlations with the ICU admission and death were recorded in patients without a history of ASCVD who received anticoagulation in therapeutic doses during hospitalization. A WHO-led meta-analysis of all trials of IL-6 antagonists confirms the benefits of Tocilizumab in severe forms of COVID-19, by reducing the risk of death by 15% [56,57]. We did not find any relationship between the immunomodulator therapy and in-hospital mortality in patients with mild or moderate forms of COVID-19 irrespective of the history of ASCVD. A possible explanation might be the small percentage of patients in our cohort who received immunomodulators.

The size and diversity of the medical conditions in subjects from our cohort, as well as the types of medical emergencies associated upon admission, contribute to the strengths of our study in characterizing the relationship between cardiac biomarker elevations, risk scores, the history of ASCVD and poor outcomes in COVID-19 patients. Thus, it is reasonable to include these cardiac biomarkers and risk scores in the patient’s diagnosis, triaging, treatment, and prognosis.

There are several limitations of this study that should be acknowledged. This was a prospective study focused on consecutive COVID-19 patients with mild and moderate forms hospitalized in a single tertiary emergency Eastern European hospital. Thus, our population should not be considered as representative for all COVID-19 patients. Larger international studies are warranted to confirm these results for patients with atherosclerotic vascular disease and COVID-19. We did not address multiethnicity in our study given the lack of ethnic diversity in our cohort. We had a low number of infections recorded for the Beta and Omicron variants, which could lead to imprecise estimates of biomarker performance in patients infected with these viral strains. Moreover, we had a very low number of vaccinated patients to be able to quantify the importance of biomarkers in this setting. Finally, we had a limited number of serial biomarker determination in our cohort, to estimate the impact of biomarkers evolution on mortality. We recommend further study to assess if serial sampling of NT-pro BNP, troponin I and D-dimer represents a better investigator of disease survival time and mortality rate in patients with ASCVD and COVID-19.

## 5. Conclusions

To our knowledge, this is the first report regarding the prognostic value of cardiac biomarkers and risk scores concerning COVID-19 patients in relation with the history of atherosclerotic cardiovascular disease. Our study supports the utility of cardiac biomarkers for the outcome prediction in patients with ASCVD and mild or moderate COVID-19. NT-pro BNP and troponin remain strong prognostic indicators of ICU admission and in-hospital death in patients with ASCVD admitted for non-severe COVID-19. Moreover, NT-pro BNP is strongly and independently associated with mortality in COVID-19 patients without a history of ASCVD. We consider that these findings may contribute not only to better management of this population, but also to a more profound understanding of the impact that COVID-19 has on the cardiovascular system. As part of a complex evaluation, along with clinical risk scores, cardiac biomarkers should be part of diagnosis evaluation in all patients with atherosclerotic cardiovascular disease and COVID-19.

## Figures and Tables

**Table 1:** Demographic, clinical and laboratory characteristics upon admission in all patients, and by history of Atherosclerotic Cardiovascular Disease (ASCVD) and composite outcome.

Variable	Total Patients (N = 1066)	Patients without ASCVD (N = 705)			Patients with ASCVD (N = 361)		
		Survivors (N = 409)	ICU/Death (N = 296)	*p*-Value	Survivors (N = 195)	ICU/Death (N = 166)	*p*-Value
Age (years) *	70 (60–78)	65 (53–73)	70 (62–79)	<0.001	72 (65–80)	76 (68–82)	0.020
Viral strain, N (%) ^#^				<0.001			<0.001
Alpha	509 (47.7)	272 (66.5)	83 (28.0)		99 (50.8)	55 (33.1)	
Beta	120 (11.3)	39 (9.5)	39 (13.2)		21 (10.8)	21 (12.7)	
Delta	349 (32.7)	69 (16.9)	166 (56.1)		34 (17.4)	80 (48.2)	
Omicron	88 (8.3)	29 (7.1)	8 (2.7)		41 (21.0)	10 (6.0)	
Male gender, N (%) ^#^	542 (51.3)	202 (49.4)	159 (53.7)	0.285	93 (47.7)	89 (53.6)	0.291
Urban residence, N (%) ^#^	576 (54.0)	197 (48.2)	182 (61.5)	0.001	102 (52.3)	95 (57.2)	0.396
Smoking, N (%) ^#^	187 (17.5)	74 (18.1)	39 (13.2)	0.096	46 (23.6)	28 (16.9)	0.119
BMI ≥25 kg/m^2^, N (%) ^#^	550 (51.6)	186 (45.5)	172 (58.1)	0.001	94 (48.2)	98 (59.0)	0.045
History HF, N (%) ^#^				0.301			<0.001
No HF	708 (66.4)	307 (75.1)	232 (78.4)		72 (36.9)	97 (58.4)	
HFpEF	172 (16.2)	51 (12.5)	26 (8.8)		66 (33.8)	29 (17.5)	
HFrEF	186 (17.4)	51 (12.5)	38 (12.8)		57 (29.2)	40 (24.1)	
History CKD, N (%) ^#^	244 (22.9)	49 (12.0)	80 (27.0)	<0.001	46 (23.6)	69 (41.6)	<0.001
History DM, N (%) ^#^	320 (30.0)	127 (31.1)	85 (28.7)	0.560	54 (27.7)	54 (32.5)	0.357
CCI score *	4 (2–5)	3 (1–4)	3 (2–5)	0.001	5 (3–6)	5 (4–6)	0.771
NEWS2 score *	6 (4–8)	5 (3–7)	7 (5–9)	<0.001	5 (3–7)	6 (4–9)	<0.001
CoLACD score *	4 (3–4)	3 (2–4)	4 (3–4)	<0.001	4 (3–5)	4 (3–5)	0.514
SaO_2_ < 90%, N (%) ^#^	356 (33.4)	64 (15.7)	172 (58.3)	<0.001	31 (16.0)	89 (53.6)	<0.001
SBP (mmHg) *	131 (120–150)	130 (120–145)	130 (114–150)	0.999	136 (120–154)	133 (117–148)	0.062
HR (bpm) *	85 (75–100)	83 (75–95)	90 (80–100)	<0.001	83 (75–95)	88 (76–100)	<0.046
GOT (U/L) *	39 (25–65)	33 (22–53)	51 (32–93)	<0.001	30 (22–48)	45 (30–75)	<0.001
Creatinine (mg/dl) *	0.9 (0.8–1.2)	0.8 (0.7–1.1)	1.0 (0.8–1.3)	<0.001	0.9 (0.8–1.2)	1.1 (0.8–1.6)	<0.001
Hemoglobin (g/dl) *	13 (11.3–14.2)	13.1 (11.4–14.2)	13.2 (11.4–14.4)	0.353	12.7 (10.9–13.8)	12.9 (11.7–14.1)	0.253
WBC (×1000/microL) *	8 (5.7–11.2)	6.9 (5.0–9.6)	8.8 (6.3–12.9)	<0.001	7.9 (5.7–10.2)	9.9 (7.0–13.8)	<0.001
CRP (mg/dL) *	6.5 (2.1–15)	4.4 (1.3–10.2)	12.1 (5.9–21.3)	<0.001	3.3 (1.1–7.9)	10.7 (4.2–19.1)	<0.001
NT-pro BNP (pg/mL) *	923 (250–3432)	499 (128–1560)	1172 (309–3344)	<0.001	1243 (377–3837)	2078 (775–7877)	0.003
hs-TnI (ng/L) *	9.5 (1.9–38.9)	5.9 (1.6–20.6)	15.9 (2.8–61.5)	0.001	9.0 (2.3–26.9)	23.1 (5.4–83.8)	0.003
D-dimer (mcg/mL) *	1.4 (0.7–3.4)	1.2 (0.6–2.7)	1.8 (0.9–4.5)	<0.001	1.2 (0.6–3.3)	1.6 (0.8–5.0)	0.037
LDH (U/L) *	284 (210–448)	247 (196–342)	458 (288–674)	<0.001	246 (202–335)	390 (218–569)	<0.001
Presepsin (pg/mL) *	363 (196–701)	280 (158–531)	604 (309–588)	<0.001	296 (179–464)	570 (370–1391)	<0.001
Abnormal ECG, N (%) ^#^ ST/T changes Dysrhythmias	74 (6.9) 493 (46.2)	19 (4.6) 163 (39.9)	24 (8.1) 138 (46.6)	0.013	15 (7.7) 109 (55.9)	16 (9.6) 83 (50.0)	0.510
Abnormal chest CT, N (%) ^#^	746 (70.0)	251 (61.4)	249 (84.1)	<0.001	115 (59.0)	131 (78.9)	<0.001

* Data are presented as the median (IQR); ^#^, % of subgroup patients; BMI, body mass index; HF, heart failure; HFpEF, heart failure with preserved ejection fraction; HFrEF, heart failure with reduced ejection fraction; CKD, chronic kidney disease; DM, diabetes mellitus; CCI, Charlson Comorbidity Index; NEWS, National Early Warning Score; CoLACD, COVID-19, Lymphocyte ratio, Age, CCI score, Dyspnea; SaO_2_, oxygen saturation; SBP, systolic blood pressure; HR, heart rate; GOT, glutamic-oxaloacetic transaminase; WBC, white blood cell count; CRP, C-reactive protein; NT-pro BNP, N terminal pro-B type natriuretic peptide; hs-TnI, high-sensitivity troponin I; LDH, lactic dehydrogenase; ECG, electrocardiogram; ST/T, ST segment/T wave; CT, computertomography.

**Table 2:** Patients’ reason for admission, treatment and outcome characteristics by history of Atherosclerotic Cardiovascular Disease (ASCVD) and composite outcome.

Variable	Total Patients (N = 1066)	Patients without ASCVD (N = 705)			Patients with ASCVD (N = 361)		
		Survivors (N = 409)	ICU/Death (N = 296)	*p*-Value	Survivors (N = 195)	ICU/Death (N = 166)	*p*-Value
Main diagnosis, N (%) ^#^							
New onset AF	15 (1.4)	6 (1.5)	4 (1.4)	0.583	2 (1.0)	3 (1.8)	0.425
Another CV emergency	343 (32.2)	88 (21.5)	54 (18.2)	0.297	108 (55.4)	93 (56.0)	0.916
HT emergency	572 (53.7)	223 (54.5)	166 (56.1)	0.702	109 (55.9)	74 (44.6)	0.035
Metabolic emergency	161 (15.1)	55 (13.4)	46 (15.5)	0.447	34 (17.4)	26 (15.7)	0.673
Liver cirrhosis/LF	38 (3.6)	15 (3.7)	13 (4.4)	0.383	5 (2.6)	5 (3.0)	0.522
Acute pancreatitis	18 (1.7)	12 (2.9)	3 (1.0)	0.112	1 (0.5)	2 (1.2)	0.596
COPD/acute asthma	62 (5.8)	19 (4.6)	13 (4.4)	0.513	16 (8.2)	14 (8.4)	0.543
Other infection	73 (6.8)	33 (8.1)	18 (6.1)	0.377	13 (6.7)	9 (5.4)	0.665
Complications, N (%) ^#^	869 (82.3)	298 (73.8)	283 (95.6)	<0.001	130 (67.7)	158 (95.8)	<0.001
MACEs, N (%) ^#^	73 (6.8)	3 (0.7)	39 (13.2)	<0.001	6 (3.1)	25 (15.1)	<0.001
H-F oxygen (NC), N (%) ^#^	468 (43.9)	183 (44.7)	114 (38.5)	0.057	83 (42.6)	88 (53.0)	0.030
CPAP or noninvasive positive pressure, N (%) ^#^	150 (14.1)	12 (2.9)	104 (35.1)	<0.001	6 (3.1)	28 (16.9)	<0.001
MV, N (%)	439 (41.2)	74 (18.1)	215 (72.6)	<0.001	34 (17.4)	116 (69.9)	<0.001
Anticoagulation, N (%) ^#^				<0.001			0.037
Prophylactic dose	432 (40.5)	188 (46.9)	119 (40.2)		57 (29.2)	68 (41.0)	
Therapeutic dose	505 (47.4)	142 (34.7)	155 (52.4)		119 (61.0)	89 (53.6)	
Treatment, N (%) ^#^							
Corticotherapy	604 (56.7)	206 (88.4)	217 (98.2)	<0.001	76 (68.5)	105 (92.9)	<0.001
Antivirals	143 (13.4)	75 (18.3)	42 (14.2)	0.087	14 (4.9)	12 (7.2)	0.253
Immunomodulators	126 (12.9)	37 (9.0)	35 (11.8)	0.257	19 (14.1)	35 (25.4)	0.023
Hospitalization (days) *	12 (4–16)	14 (11–17)	4 (2–10)	<0.001	15 (12–18)	5 (2–11)	<0.001

^#^ Data are presented as the % of subgroup patients; * data are presented as the median (IQR); AF, atrial fibrillation; CV, cardiovascular; HT, hypertensive; LF, liver failure; COPD, chronic obstructive pulmonary disease; MACEs, major adverse cardiovascular events; H-F, high flow; NC, nasal cannula; ICU, intensive care unit; CPAP, continuous positive airway pressure; MV, mechanical ventilation.

**Table 3:** Cox proportional hazards model assessing the relationship between N-terminal B-type natriuretic peptide and the main outcome adjusted for multiple relevant covariates, in relation with history of ASCVD.

	Patients without ASCVD				Patients with ASCVD			
	Univariate		Multivariate		Univariate		Multivariate	
	HR (95% CI)	*p* Value	HR (95% CI)	*p* Value	HR (95% CI)	*p* Value	HR (95% CI)	*p* Value
NT-pro BNP *	1.70 (1.22–2.38)	0.002	1.66 (1.10–2.53)	0.016	2.37 (1.56–3.59)	<0.001	2.63 (1.65–4.18)	<0.001
Strain (Delta)	2.51 (0.90–6.95)	0.078	7.45 (0.90–61.48)	0.062	2.32 (1.08–4.99)	0.031	4.82 (1.06–22.01)	0.042
HT emergency	1.22 (0.78–1.90)	0.099	1.07 (0.59–1.97)	0.182	1.87 (1.09–3.20)	0.024	2.08 (1.03–4.21)	0.042
CCI score	1.17 (1.07–1.29)	0.001	1.07 (0.91–1.26)	0.397	1.10 (0.99–1.22)	0.081	1.13 (0.96–1.33)	0.134
NEWS2 score > 5	1.68 (1.07–2.64)	0.023	0.69 (0.35–1.34)	0.268	1.16 (0.72–1.88)	0.053	1.55 (0.79–3.03)	0.205
CoLACD score	1.14 (0.97–1.34)	0.098	1.12 (0.90–1.40)	0.214	1.11 (0.87–1.41)	0.094	1.13 (0.85–1.51)	0.384
SaO_2_ > 90%	0.22 (0.14–0.33)	<0.001	0.42 (0.22–0.79)	0.007	0.31 (0.20–0.49)	<0.001	0.55 (0.27–1.10)	0.090
hs-CRP	1.05 (1.03–1.06)	<0.001	1.01 (0.97–1.04)	0.678	1.04 (1.03–1.06)	<0.001	1.04 (1.01–1.07)	0.009
BMI < 25 kg/m^2^	0.57 (0.38–0.85)	0.006	0.82 (0.45–1.47)	0.497	0.46 (0.29–0.75)	0.002	0.50 (0.26–0.96)	0.037
CKD	0.40 (0.26–0.62)	<0.001	0.69 (0.36–1.34)	0.278	0.51 (0.32–0.80)	0.003	1.62 (0.79–3.35)	0.192
Anticoagulation **	0.56 (0.27–1.18)	0.017	0.63 (0.40–1.00)	0.052	0.44 (0.11–1.82)	0.098	0.79 (0.45–1.37)	0.401
Age >65 years	0.42 (0.26–0.67)	<0.001	0.62 (0.32–1.19)	0.150	0.87 (0.51–1.50)	0.086	1.29 (0.60–2.80)	0.519

ASCVD, atherosclerotic cardiovascular disease; HR, hazard ratio; CI, confidence interval; *, per logarithmic unit; NT-pro BNP, N-terminal pro B-type natriuretic peptide; HT, hypertensive; CCI, Charlson Comorbidity Index; NEWS, National Early Warning Score; CoLACD, COVID-19, Lymphocyte ratio, Age, CCI score, Dyspnea; SaO_2_, oxygen saturation upon admission; hs-CRP, high-sensitivity C-reactive protein; BMI, body mass index; CKD, chronic kidney disease; **, reference therapeutic dose.

**Table 4:** Cox proportional hazards model assessing the relationship between admission high-sensitivity troponin I and the main outcome adjusted for multiple relevant covariates, in relation with history of ASCVD.

	**Patients without ASCVD**				**Patients with ASCVD**			
	**Univariate**		**Multivariate**		**Univariate**		**Multivariate**	
	**HR (95% CI)**	***p* Value**	**HR (95% CI)**	***p* Value**	**HR (95% CI)**	***p* Value**	**HR (95% CI)**	***p* Value**
hs-TnI *	1.26 (0.95–1.68)	0.092	1.09 (0.84–1.43)	0.522	1.31 (1.03–1.65)	0.025	1.51 (1.13–2.03)	0.006
HT emergency	1.22 (0.78–1.90)	0.099	0.98 (0.47–2.03)	0.947	1.87 (1.09–3.20)	0.024	2.94 (1.19–7.28)	0.020
Strain (Delta)	2.51 (0.90–6.95)	0.078	2.63 (0.55–12.56)	0.226	2.32 (1.08–4.99)	0.031	1.17 (0.26–5.25)	0.835
CCI score	1.17 (1.07–1.29)	0.001	1.21 (0.99–1.49)	0.064	1.10 (0.99–1.22)	0.081	1.20 (0.99–1.46)	0.069
NEWS2 score > 5	1.68 (1.07–2.64)	0.023	0.52 (0.21–1.34)	0.176	1.16 (0.72–1.88)	0.053	1.69 (0.75–3.82)	0.210
CoLACD score	1.14 (0.97–1.34)	0.098	1.17 (0.90–1.52)	0.236	1.11 (0.87–1.41)	0.094	1.07 (0.76–1.51)	0.686
SaO_2_ > 90%	0.22 (0.14–0.33)	<0.001	0.44 (0.21–0.92)	0.030	0.31 (0.20–0.49)	<0.001	0.22 (0.09–0.57)	0.002
hs-CRP	1.05 (1.03–1.06)	<0.001	1.05 (1.01–1.08)	0.015	1.04 (1.03–1.06)	<0.001	1.07 (1.03–1.11)	<0.001
BMI < 30 kg/m^2^	0.57 (0.38–0.85)	0.006	1.21 (0.59–2.51)	0.600	0.46 (0.29–0.75)	0.002	0.93 (0.37–2.34)	0.880
CKD	0.40 (0.26–0.62)	<0.001	0.68 (0.33–1.41)	0.299	0.51 (0.32–0.80)	0.003	1.67 (0.73–3.84)	0.228
Anticoagulation **	0.56 (0.27–1.18)	0.017	0.51 (0.29–0.90)	0.020	0.44 (0.11–1.82)	0.098	0.54 (0.28–1.06)	0.072
Age < 65 years	0.42 (0.26–0.67)	<0.001	0.71 (0.33–1.54)	0.380	0.87 (0.51–1.50)	0.086	0.69 (0.26–1.83)	0.454

ASCVD, atherosclerotic cardiovascular disease; HR, hazard ratio; CI, confidence interval; *, per logarithmic unit; hs-TnI, high-sensitivity troponin I; HT, hypertensive; CCI, Charlson Comorbidity Index; NEWS, National Early Warning Score; CoLACD, COVID-19, Lymphocyte ratio, Age, CCI score, Dyspnea; SaO_2_, oxygen saturation upon admission; hs-CRP, high-sensitivity C-reactive protein; BMI, body mass index; CKD, chronic kidney disease; ** reference therapeutic dose.

## Data Availability

The data presented in this study are available in this article and Supplementary Materials.

## Supplementary Materials

The following supporting information can be downloaded at: https://www.mdpi.com/article/10.3390/jcm11195671/s1, Figure S1: Flowchart of the study. Table S1: Cardiac biomarkers upon admission showed significant differences based on the history of ASCVD and viral strain involved. Figure S2: Significant correlations between NT-pro BNP quartiles and hs-TnI: (**a**) hs-TnI per log unit is correlated with NT-pro BNP quartiles in patients with no history of ASCVD; (**b**) NT-pro BNP quartiles are correlated with hs-TnI (per log. unit) in patients with history of ASCVD. Figure S3: Correlation between NT-pro BNP quartiles and D-dimers: (**a**) D-dimers per log unit are correlated with NT-pro BNP quartiles in patients without history of ASCVD; (**b**) no statistically significant correlation between D-dimers per log unit and NT-pro BNP quartiles in patients with ASCVD. Table S2: Cox proportional hazards model assessing the relationship between N-terminal B-type natriuretic peptide and the main outcome adjusted for multiple relevant covariates, including high-sensitivity troponin I, in relation with the history of ASCVD. Table S3: Cox proportional hazards model assessing the relationship between N-terminal B-type natriuretic peptide and the main outcome adjusted for multiple relevant covariates, including D-dimers, in relation with the history of ASCVD. Table S4: Model assessing the relationship between N-terminal pro B-type natriuretic peptide and MACEs adjusted for multiple relevant covariates, based on the history of ASCVD. Figure S4: Boxplots revealing significant correlations between admission hs-TnI and the main composite outcome: (**a**) In patients without a history of ASCVD; (**b**) In patients with ASCVD. °, represent outliers. Table S5: Model assessing the relationship between hs-TnI and MACEs adjusted for multiple relevant covariates, based on the history of ASCVD. Table S6: Cox proportional hazards model assessing the relationship between N-terminal B-type natriuretic peptide and the main outcome adjusted for multiple relevant covariates, including D-dimers, and hs-TnI, in relation with the history of ASCVD. Figure S5: Receiver operating characteristic (ROC) curve assessed the discriminatory ability of the adjusted models including classical cardiovascular risk factors predictive for composite endpoint ICU admission and death in patients with mild-to-moderate COVID-19 and ASCVD (model 1), to which were added NT-proBNP (model 2), hs-TnI (model 3), NT-proBNP and D-dimer (model 4), and NT-proBNP with D-dimer and hs-TnI (model 5).
