# Supplementary material for: Cardiac Biomarkers and Risk Scores in Relation with History of Atherosclerotic Cardiovascular Disease in Patients Admitted with COVID-19: The Experience of an Eastern European Center
Source: J Clin Med. 2022 Sep 26;11(19):5671. doi: 10.3390/jcm11195671 (PMC9571947; doi:10.3390/jcm11195671)
Supplement: Supplementary file 1 [file jcm-11-05671-s001.zip › jcm-1912660-supplementary.pdf]

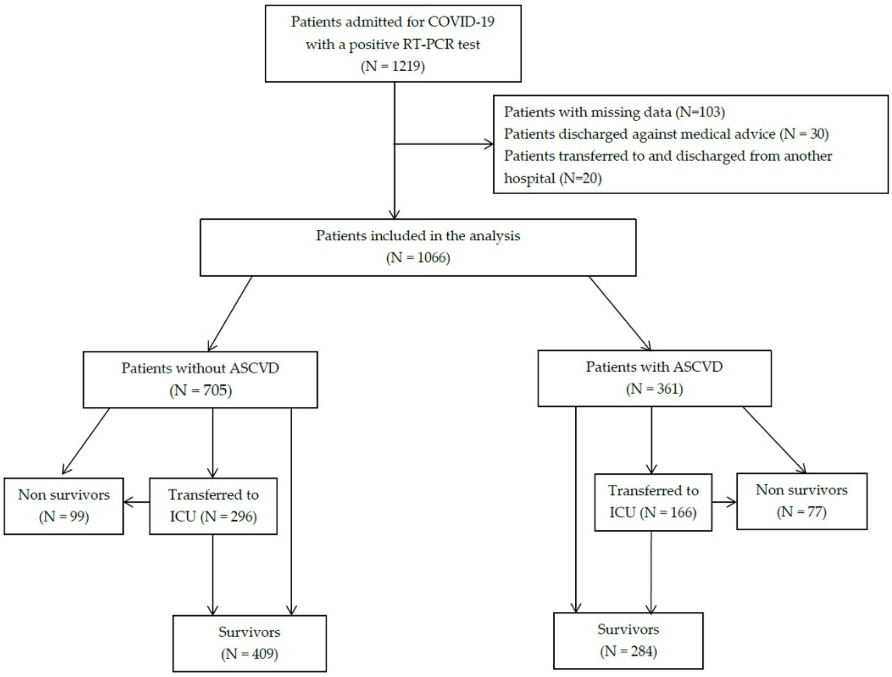

Figure S1. Flowchart of the study.

Table S1. Cardiac biomarkers upon admission showed significant differences based on the history of ASCVD and viral strain involved.

Viral strain	Biomarker	Without history of ASCVD	With ASCVD	p-value
Alpha	NT-pro BNP*	670 [185-2492]	1675 [713-3831]	< 0.001
	hs-TnI*	7.2 [2.3-30.7]	13.9 [5.6-67.9]	0.024
Beta	NT-pro BNP	525 [97-3068]	1217 [547-7673]	0.027
	hs-TnI	3.0 [0.1-14.6]	11.6 [0.1-36.9]	0.122
Delta	NT-pro BNP	872 [253-3445]	1466 [594-5850]	0.003
	hs-TnI	13.7 [3.2-57.4]	17.1 [3.0-56.5]	0.711
Omicron	NT-pro BNP	2445 [668-4426]	2311 [939-9188]	0.485
	hs-TnI	13.6 [1.4-40.6]	10.5 [4.0-35.6]	0.748

* Values expressed as median [IQR].

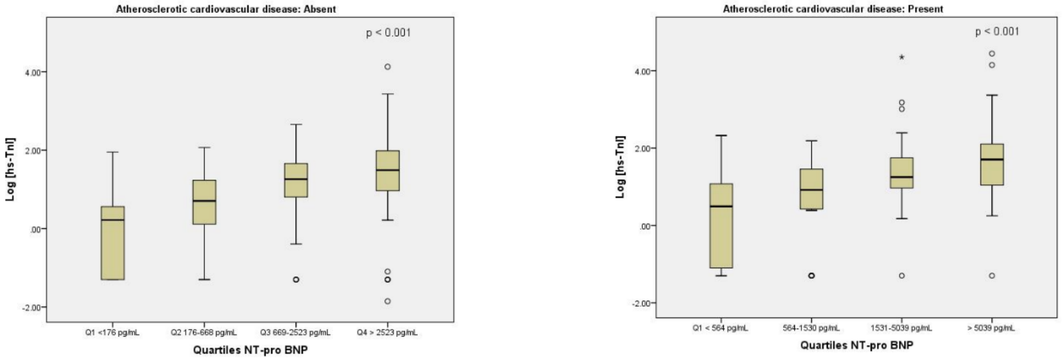

(**a**) (**b**)

Figure S2. Significant correlations between NT-pro BNP quartiles and hs-TnI: (**a**) hs-TnI per log unit is correlated with NT-pro BNP quartiles in patients with no history of ASCVD; (**b**) NT-pro BNP quartiles are correlated with hs-TnI (per log. unit) in patients with history of ASCVD. °, represent outliers; *, represent extreme values.

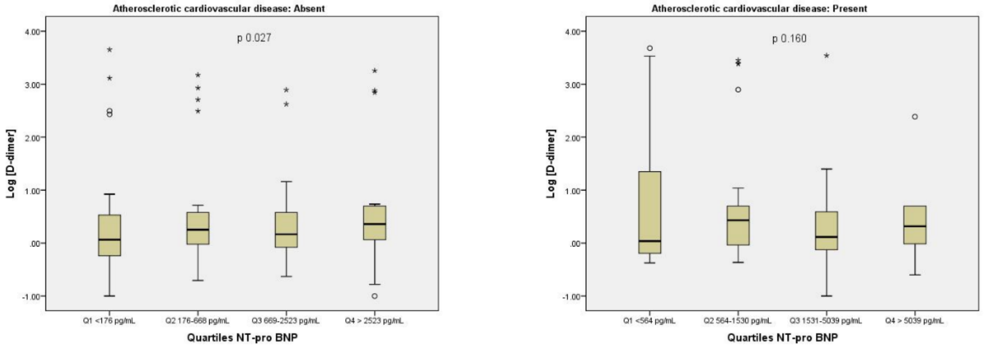

(**a**) (**b**)

Figure S3. Correlation between NT-pro BNP quartiles and D-dimers: (**a**) D-dimers per log unit are correlated with NT-pro BNP quartiles in patients without history of ASCVD; (**b**) no statistically significant correlation between D-dimers per log unit and NT-pro BNP quartiles in patients with ASCVD. °, represent outliers; *, represent extreme values.

Table S2. Cox proportional hazards model assessing the relationship between N-terminal B-type natriuretic peptide and the main outcome adjusted for multiple relevant covariates, including high sensitivity troponin I, in relation with the history of ASCVD.

	Patients without ASCVD				Patients with ASCVD			
	Univariate		Multivariate		Univariate		Multivariate	
	HR (95%CI)	P value	HR (95%CI)	P value	HR (95%CI)	P value	HR (95%CI)	P value
NT-pro BNP*	1.70 (1.22-2.38)	0.002	1.62 (1.08-2.43)	0.020	2.37 (1.56-3.59)	<0.001	2.69 (1.68-4.29)	< 0.001
Strain (Delta)	2.51 (0.90-6.95)	0.078	7.22 (0.90-57.87)	0.063	2.32 (1.08-4.99)	0.031	6.87 (1.35-35.07)	0.020
HT emergency	1.22 (0.78-1.90)	0.099	0.98 (0.47-2.03)	0.947	1.87 (1.09-3.20)	0.024	2.94 (1.19-7.28)	0.020
CCI score	1.17 (1.07-1.29)	0.001	1.05 (0.89-1.24)	0.563	1.10 (0.99-1.22)	0.081	1.11 (0.95-1.29)	0.195
NEWS2 score > 5	1.68 (1.07-2.64)	0.023	0.69 (0.36-1.36)	0.284	1.16 (0.72-1.88)	0.053	1.31 (0.67-2.56)	0.426
CoLACD score	1.14 (0.97-1.34)	0.098	1.12 (0.90-1.40)	0.312	1.11 (0.87-1.41)	0.094	1.12 (0.83-1.50)	0.454
SaO2 > 90%	0.22 (0.14-0.33)	<0.001	0.37 (0.19-0.70)	0.002	0.31 (0.20-0.49)	< 0.001	0.63 (0.31-1.27)	0.195
hs-CRP	1.05 (1.03-1.06)	<0.001	1.01 (0.97-1.04)	0.705	1.04 (1.03-1.06)	<0.001	1.04 (1.01-1.07)	0.011
BMI < 25 kg/m2	0.57 (0.38-0.85)	0.006	0.83 (0.46-1.49)	0.523	0.46 (0.29-0.75)	0.002	0.56 (0.29-1.08)	0.082
CKD	0.40 (0.26-0.62)	<0.001	0.71 (0.37-1.37)	0.307	0.51 (0.32-0.80)	0.003	1.37 (0.67-2.79)	0.384
Anticoagulation**	0.56 (0.27-1.18)	0.017	1.18 (0.65-2.18)	0.581	0.44 (0.11-1.82)	0.098	1.05 (0.67-1.09)	0.486
Age > 65 years	0.42 (0.26-0.67)	<0.001	0.58 (0.30-1.13)	0.112	0.87 (0.51-1.50)	0.086	2.49 (1.56-3.98)	0.200
hs-TnI*	1.26 (0.95-1.68)	0.092	2.08 (0.78-5.51)	0.142	1.31 (1.03-1.65)	0.025	2.59 (0.70-9.55)	0.153

*, per logarithmic unit; HT, hypertensive; CCI, Charlson Comorbidity Index; NEWS, National Early Warning Score; CoLACD, CoVID-19, Lymphocyte ratio, Age, CCI score, Dyspnea; SaO2, oxygen saturation upon admission; hs-CRP, high sensitivity C-reactive protein; BMI, body mass index; CKD, chronic kidney disease; **, reference therapeutic dose.

Table S3. Cox proportional hazards model assessing the relationship between N-terminal B-type natriuretic peptide and the main outcome adjusted for multiple relevant covariates, including D-dimers, in relation with the history of ASCVD.

	Patients without ASCVD				Patients with ASCVD			
	Univariate		Multivariate		Univariate		Multivariate	
	HR (95%CI)	P value	HR (95%CI)	P value	HR (95%CI)	P value	HR (95%CI)	P value
NT-pro BNP*	1.70 (1.22-2.38)	0.002	1.69 (1.09-2.63)	0.019	2.37 (1.56-3.59)	<0.001	3.34 (1.89-5.88)	< 0.001
Strain (Delta)	2.51 (0.90-6.95)	0.078	2.95 (0.35-24.66)	0.318	2.32 (1.08-4.99)	0.031	5.66 (0.84-38.26)	0.075
HT emergency	1.22 (0.78-1.90)	0.099	0.95 (0.50-1.84)	0.889	1.87 (1.09-3.20)	0.024	2.21 (0.97-5.04)	0.060
CCI score	1.17 (1.07-1.29)	0.001	1.12 (0.94-1.32)	0.207	1.10 (0.99-1.22)	0.081	1.10 (0.90-1.35)	0.335
NEWS2 score > 5	1.68 (1.07-2.64)	0.023	0.57 (0.27-1.21)	0.146	1.16 (0.72-1.88)	0.053	1.71 (0.77-3.80)	0.190
CoLACD score	1.14 (0.97-1.34)	0.098	1.03 (0.79-1.35)	0.813	1.11 (0.87-1.41)	0.094	1.18 (0.84-1.66)	0.328
SaO2 > 90%	0.22 (0.14-0.33)	<0.001	0.28 (0.14-0.58)	0.001	0.31 (0.20-0.49)	< 0.001	0.48 (0.21-1.10)	0.081
hs-CRP	1.05 (1.03-1.06)	<0.001	1.01 (0.97-1.05)	0.617	1.04 (1.03-1.06)	<0.001	1.04 (1.00-1.07)	0.051
BMI < 25 kg/m2	0.57 (0.38-0.85)	0.006	0.79 (0.41-1.50)	0.463	0.46 (0.29-0.75)	0.002	0.42 (0.20-0.89)	0.024
CKD	0.40 (0.26-0.62)	<0.001	0.60 (0.29-1.24)	0.167	0.51 (0.32-0.80)	0.003	1.57 (0.67-3.64)	0.299
Anticoagulation**	0.56 (0.27-1.18)	0.017	0.63 (0.39-1.03)	0.067	0.44 (0.11-1.82)	0.098	0.65 (0.34-1.24)	0.193
Age > 65 years	0.42 (0.26-0.67)	<0.001	0.67 (0.33-1.36)	0.267	0.87 (0.51-1.50)	0.086	1.47 (0.61-3.55)	0.397
D-dimers*	1.23 (0.89-1.68)	0.207	1.18 (0.76-1.84)	0.461	1.11 (0.82-1.51)	0.513	1.29 (0.77-2.17)	0.335

*, per logarithmic unit; HT, hypertensive; CCI, Charlson Comorbidity Index; NEWS, National Early Warning Score; CoLACD, CoVID-19, Lymphocyte ratio, Age, CCI score, Dyspnea; SaO2, oxygen saturation upon admission; hs-CRP, high sensitivity C-

reactive protein; BMI, body mass index; CKD, chronic kidney disease; **, reference therapeutic dose.

Table S4: Model assessing the relationship between N-terminal pro B-type natriuretic peptide and MACE adjusted for multiple relevant covariates, based on the history of ASCVD.

Variable	Patients without ASCVD				Patients with ASCVD			
	Univariate		Multivariate		Univariate		Multivariate	
	HR (95%CI)	P value	HR (95%CI)	P value	HR (95%CI)	P value	HR (95%CI)	P value
Age	1.01 (0.99-1.03)	0.031	1.03 (0.98-1.08)	0.311	1.00 (0.97-1.04)	0.083	0.91 (0.85-0.99)	0.021
Smoking	1.16 (0.48-2.81)	0.056	1.05 (0.21-5.23)	0.951	1.08 (0.43-2.74)	0.088	6.44 (0.70-59.16)	0.100
CCI	1.11 (0.96-1.28)	0.045	0.62 (0.40-0.96)	0.030	1.05 (0.88-1.25)	0.073	1.17 (0.83-1.66)	0.379
HT emergency	1.22 (0.61-2.42)	0.055	0.84 (0.28-2.49)	0.752	2.06 (0.86-4.93)	0.056	3.90 (0.72-21.08)	0.114
hs-CRP	1.01 (0.99-1.04)	0.058	0.99 (0.95-1.04)	0.807	1.00 (0.96-1.04)	0.089	0.95 (0.87-1.04)	0.284
Corticotherapy	2.46 (0.80-7.61)	0.060	0.85 (0.09-8.26)	0.892	0.99 (0.32-3.11)	0.100	1.45 (0.21-10.02)	0.707
NT-pro BNP*	2.72 (1.58-4.68)	< 0.001	4.61 (2.00-10.64)	<0.001	4.88 (2.21-10.78)	< 0.001	5.23 (1.64-16.62)	0.005
DM	0.51 (0.24-1.07)	0.075	0.31 (0.09-1.04)	0.059	1.04 (0.38-2.83)	0.091	0.59 (0.11-3.27)	0.548

*, per log. unit; CCI, Charlston Comorbidity index; HT, hypertensive; hs-CRP, high-sensitivity C-reactive protein; DM, diabetes mellitus.

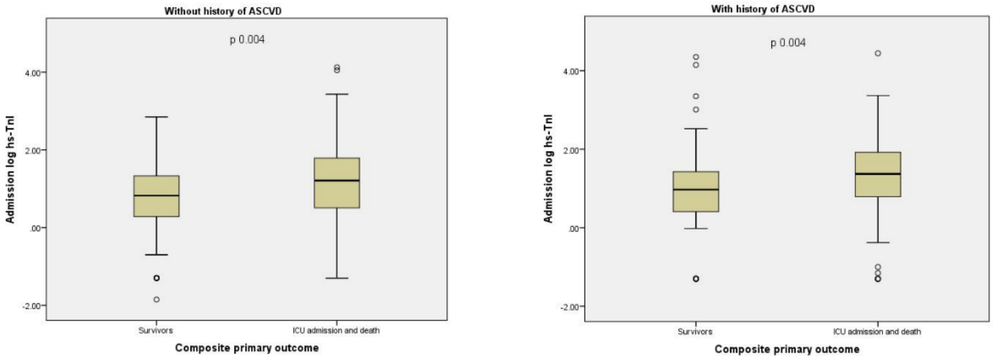

(**a**) (**b**)

Figure S4. Boxplots revealing significant correlations between admission hs-TnI and the main composite outcome: (a) In patients without a history of ASCVD; (b) In patients with ASCVD. °, represent outliers.

Table **S5**: Model assessing the relationship between hs-TnI and MACE adjusted for multiple relevant covariates, based on the history of ASCVD.

**Variable**	**Patients without ASCVD**				**Patients with ASCVD**			
	**Univariate**		**Multivariate**		**Univariate**		**Multivariate**	
	**HR (95%CI)**	**P value**	**HR (95%CI)**	**P value**	**HR (95%CI)**	**P value**	**HR (95%CI)**	**P value**
Age	1.01 (0.99-1.03)	0.031	1.00 (0.96-1.05)	0.897	1.00 (0.97-1.04)	0.083	0.96 (0.89-1.04)	0.343
Smoking	1.16 (0.48-2.81)	0.056	2.03 (0.24-17.01)	0.514	1.08 (0.43-2.74)	0.088	1.24 (0.18-8.53)	0.828
CCI	1.11 (0.96-1.28)	0.045	0.83 (0.56-1.22)	0.335	1.05 (0.88-1.25)	0.073	1.11 (0.77-1.61)	0.569
HT emergency	1.22 (0.61-2.42)	0.055	1.52 (0.51-4.50)	0.452	2.06 (0.86-4.93)	0.056	3.05 (0.55-16.75)	0.200
hs-CRP	1.01 (0.99-1.04)	0.058	1.00 (0.96-1.05)	0.885	1.00 (0.96-1.04)	0.089	0.95 (0.87-1.05)	0.312
Corticotherapy	2.46 (0.80-7.61)	0.060	0.91 (0.10-8.41)	0.933	0.99 (0.32-3.11)	0.100	2.06 (0.29-14.50)	0.469
hs-TnI*	1.60 (1.08-2.38)	0.019	1.63 (1.02-2.60)	0.042	1.95 (1.25-3.04)	0.003	3.26 (1.51-7.06)	0.003
DM	0.51 (0.24-1.07)	0.075	2.22 (0.66-7.44)	0.195	1.04 (0.38-2.83)	0.091	2.97 (0.60-14.77)	0.183

*, per log. unit; CCI, Charlston Comorbidity index; HT, hypertensive; hs-CRP, high-sensitivity C-reactive protein; hs-TnI, hgh-sensitivity troponin I; DM, diabetes mellitus.

Table S6. Cox proportional hazards model assessing the relationship between N-terminal B-type natriuretic peptide and the main outcome adjusted for multiple relevant covariates, including D-dimers, and hs-TnI, in relation with the history of ASCVD.

	**Patients without ASCVD**				**Patients with ASCVD**			
	**Univariate**		**Multivariate**		**Univariate**		**Multivariate**	
	**HR (95%CI)**	**P value**	**HR (95%CI)**	**P value**	**HR (95%CI)**	**P value**	**HR (95%CI)**	**P value**
NT-pro BNP*	1.70 (1.22-2.38)	0.002	1.82 (1.02-3.26)	0.043	2.37 (1.56-3.59)	<0.001	3.54 (1.98-6.33)	<0.001
Strain (Delta)	2.51 (0.90-6.95)	0.078	3.11 (0.34-28.33)	0.315	2.32 (1.08-4.99)	0.031	3.46 (0.37-32.14)	0.275
HT emergency	1.22 (0.78-1.90)	0.099	1.10 (0.50-2.43)	0.821	1.87 (1.09-3.20)	0.024	2.05 (0.70-5.96)	0.190
CCI score	1.17 (1.07-1.29)	0.001	1.12 (0.91-1.39)	0.276	1.10 (0.99-1.22)	0.081	1.02 (0.75-1.38)	0.906
NEWS2 score > 5	1.68 (1.07-2.64)	0.023	0.48 (0.18-1.31)	0.152	1.16 (0.72-1.88)	0.053	3.17 (1.09-9.26)	0.035
CoLACD score	1.14 (0.97-1.34)	0.098	1.09 (0.78-1.50)	0.620	1.11 (0.87-1.41)	0.094	1.29 (0.81-2.05)	0.290
SaO2 > 90%	0.22 (0.14-0.33)	<0.001	0.26 (0.11-0.64)	0.004	0.31 (0.20-0.49)	< 0.001	0.19 (0.06-0.62)	0.006
hs-CRP	1.05 (1.03-1.06)	<0.001	1.04 (0.99-1.08)	0.090	1.04 (1.03-1.06)	<0.001	1.09 (1.03-1.14)	0.001
BMI < 25 kg/m2	0.57 (0.38-0.85)	0.006	0.77 (0.35-1.71)	0.521	0.46 (0.29-0.75)	0.002	0.59 (0.18-1.92)	0.383
CKD	0.40 (0.26-0.62)	<0.001	0.74 (0.31-1.76)	0.490	0.51 (0.32-0.80)	0.003	3.15 (1.01-9.88)	0.049
Anticoagulation**	0.56 (0.27-1.18)	0.017	0.43 (0.23-0.80)	0.007	0.44 (0.11-1.82)	0.098	0.47 (0.17-1.27)	0.135
Age > 65 years	0.42 (0.26-0.67)	<0.001	0.52 (0.22-1.21)	0.131	0.87 (0.51-1.50)	0.086	1.88 (0.46-7.73)	0.381
D-dimers*	1.23 (0.89-1.68)	0.207	1.26 (0.75-2.13)	0.386	1.11 (0.82-1.51)	0.513	1.95 (0.91-4.21)	0.087
hs-TnI*	1.26 (0.95-1.68)	0.092	0.95 (0.69-1.32)	0.773	1.31 (1.03-1.65)	0.025	2.52 (1.39-4.56)	0.002

*, per logarithmic unit; HT, hypertensive; CCI, Charlson Comorbidity Index; NEWS, National Early Warning Score; CoLACD, CoVID-19, Lymphocyte ratio, Age, CCI score, Dyspnea; SaO2, oxygen saturation upon admission; hs-CRP, high sensitivity C-reactive protein; BMI, body mass index; CKD, chronic kidney disease; **, reference therapeutic dose.

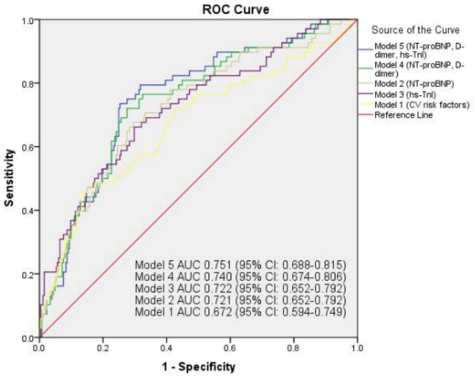

Figure S5. Receiver operating characteristic (ROC) curve assessed the discriminatory ability of the adjusted models including classical cardiovascular risk factors predictive for composite end-point ICU admission and death in patients with mild-to-moderate COVID-19 and ASCVD (model 1), to which were added NT-proBNP (model 2), hs-TnI (model 3), NT-proBNP and D-dimer (model 4), and NT-proBNP with D-dimer hs-TnI (model 5).
